# NF-Kappa B Modulation Is Involved in Celastrol Induced Human Multiple Myeloma Cell Apoptosis

**DOI:** 10.1371/journal.pone.0095846

**Published:** 2014-04-22

**Authors:** Haiwen Ni, Wanzhou Zhao, Xiangtu Kong, Haitao Li, Jian Ouyang

**Affiliations:** 1 Affiliated Hospital of Nanjing University of TCM, Nanjing, China; 2 Sino-EU Biomedical Innovation Center (SEBIC), OG Pharma Corporation, Nanjing, China; 3 Nanjing University of Chinese Medicine, Nanjing,China; 4 Department of Hematology, Nanjing Drum Tower Hospital, the Affiliated Hospital of Nanjing University Medical School, Nanjing, China; Duke University Medical Center, United States of America

## Abstract

Celastrol is an active compound extracted from the root bark of the traditional Chinese medicine *Tripterygium wilfordii Hook F*. To investigate the effect of celastrol on human multiple myeloma cell cycle arrest and apoptosis and explore its molecular mechanism of action. The activity of celastrol on LP-1 cell proliferation was detected by WST-8 assay. The celastrol-induced cell cycle arrest was analyzed by flow cytometry after propidium iodide staining. Nuclear translocation of the nuclear factor kappa B (NF-κB) was observed by fluorescence microscope. Celastrol inhibited cell proliferation of LP-1 myeloma cell in a dose-dependent manner with IC50 values of 0.8817 µM, which was mediated through G1 cell cycle arrest and p27 induction. Celastrol induced apoptosis in LP-1 and RPMI 8226 myeloma cells in a time and dose dependent manner, and it involved Caspase-3 activation and NF-κB pathway. Celastrol down-modulated antiapoptotic proteins including Bcl-2 and survivin expression. The expression of NF-κB and IKKa were decreased after celastrol treatment. Celastrol effectively blocked the nuclear translocation of the p65 subunit and induced human multiple myeloma cell cycle arrest and apoptosis by p27 upregulation and NF-kB modulation. It has been demonstrated that the effect of celastrol on NF-kB was HO-1-independent by using zinc protoporphyrin-9 (ZnPPIX), a selective heme oxygenase inhibitor. From the results, it could be inferred that celastrol may be used as a NF-kB inhibitor to inhibit myeloma cell proliferation.

## Introduction

Multiple myeloma (MM) is still an incurable hematological malignancy with a median survival of 4 years despite the use of various treatment options including thalidomide, lenalidomide, bortezomib, and hematopoietic stem cell transplantation [1], [2]. The findings in molecular mechanisms that lead to MM and its progression have lead to the clarification of molecular targets of this disease and may contribute to the development of new biological targeted therapies for MM [3].

MM is a fatal plasma cell malignancy arising from the mature plasma cells in the bone marrow characterized by bone destruction, hypercalcemia, anemia, immunodeficiency, and renal damage [4]. The patients suffering from MM often result in recurrent or increased susceptibility to bacterial, fungal, and viral infections which remain a major cause of their deaths [5], [6].

For the past 30 years, many natural products derived from plants and marine have provided leading structures for developing new agents with enhanced biological properties and less toxicity than chemotherapeutic agents [7], [8].

Many natural products induced apoptosis of human cancer cells through the basic molecular mechanisms that take place in cancer [9], [10]. It has been reported that the cancer and inflammation may have common signal pathways [11]–[14]. It is our hypothesis that the novel therapeutic agents with anti-inflammatory activity may prolong MM progression and overcome drug resistance.

Celastrol is an active compound extracted from the root bark of the traditional Chinese medicine Tripterygium wilfordii Hook F [15]–[18]. It has been effectively used in the treatment of chronic inflammation and autoimmune diseases such as arthritis, lupus erythematosus, and lateral sclerosis [19], [20]. Although celastrol was reported to inhibit multiple cancer cell proliferation and induce cell death such as breast cancer [21], colon cancer [22], prostate cancer [23], [24], oral squamous cell carcinoma [25], glioma [26], melanoma [27], and leukemia [28], the direct targets and molecular mechanisms of celastrol-induced apoptosis in cancer cells remain unknown. In present the study, an attempt to investigate the effect of celastrol on LP-1 human myeloma cell apoptosis and its molecular mechanism of action was made.

## Material and Methods

### 2.1. Reagents

A 100 mM solution of celastrol (from Sigma) was prepared in dimethyl sulfoxide (DMSO) and stored as small aliquots at −20°C. Subsequent dilutions were made in a cell culture medium. The same proportion of DMSO/culture medium was added to the controls. The final DMSO content was less than 0.1%. Penicillin, streptomycin, Dulbecco's modified Eagle's medium, Rosewell Park Memorial Institute (shortly RPMI-1640) medium, and fetal bovine serum were obtained from Invitrogen. Propidium Iodide/Ribonuclease (shortly PI/RNase) Staining Buffer and Annexin V-Fluorescein Isothiocyanate (FITC) Apoptosis Detection Kit I were purchased from BD Pharmingen (USA).

### 2.2. Cell line and culture conditions

Human MM cell line LP-1 (Deutsche Sammlung von Mikroorganismen und Zellkulturen GmbH, Germany) was cultured in Iscove's modified Dulbecco's medium (Gibco), and RPMI 8226 (ATCC) was cultured in RPMI 1640 medium (Gibco) containing 10% fetal calf serum, 2 mmol/L l-glutamine, and 100 U/mL penicillin, and 100 mg/mL streptomycin. All cell lines were maintained at 37°C in a fully humidified atmosphere of 5% carbondioxide in air.

### 2.3. Cell viability assay [29]

The antiproliferative effects of celastrol on MM cells were determined by the WST-8 dye uptake method. The cells were plated into a 96-well plate at a density of 1×104/well and treated with the indicated dose of celastrol for 72 hours of incubation. For the cell viability assay, 20 µL of WST-8 solution (EnoGene) was added to each well. The plates were further incubated for 4 hours. The absorbance of each well was measured using an enzyme-linked immunosorbent assay reader at 450 nm.

### 2.4. Deoxyribonucleic acid (DNA) content and cell cycle analysis [30]

The celastrol-treated cells were fixed in 70% ethanol at 4°C overnight. After twice washed with phosphate buffer solution (PBS), the cells were suspended in hypotonic solution containing 0.1% Triton X-100, 1 mM Tris-hydrochloride (pH 8.0), 3.4 mM sodium citrate, 0.1 mM EDTA; and the cells were stained with PI (50 µg/mL) and RNase A (1 mg/mL) for 30 min. A total of 10,000 events were analyzed by flow cytometry using an excitation wavelength set at 488 nm and emission set at 610 nm. The DNA content was analyzed by flow cytometry (FACScalibur, BD Biosciences). The population of cells in each cycle phase was analyzed using WinMDI 2.8 software (Purdue University Cytometry Laboratory).

### 2.5. Annexin V apoptosis assay [31]

The cells were treated with celastrol (0.25 and 0.5 µM) for the indicated times (0–48 h). The apoptotic cells were determined by double staining with Annexin V-FITC and PI according to the instructions of kit's manufacturer (EnoGene). The cells were analyzed on a flow cytometer (FACSCalibur, Becton-Dickinson, San Jose, CA) using CellQuest software. The cells that were Annexin V (−) and PI (−) were considered viable cells; Annexin V (+) and PI (−) were considered early-stage apoptotic cells; Annexin V (+) and PI (+) were considered late-stage apoptotic cells; and Annexin V (−) and PI (+) were considered necrotic cells.

### 2.6. Caspase activation assay [32]

Caspase-3 activity was determined by using a caspase-3 assay kit (PharMingen, San Diego, CA) according to the manufacturer's instructions. The cells (1×105) were homogenized in a lysis buffer containing 0.1% CHAPS, 50 mM HEPES (pH 8.0), 12.5 mM NaCl, 0.1 mM EDTA, and 5 mM DTT freshly added. The whole cell lysates (40 mg) were incubated with 20 mM of colorimetric substrate acetyl-Asp-Glu-Val-Asp p-nitroanilide (Ac-DEVD-pNA) in a reaction buffer (BioVision) containing 5 mM DTT at 37°C for 1 h. pNA was released from the substrate upon cleavage by DEVDase. The yellow color produced by free pNA was monitored by a spectrophotometer at 405 nm. The amount of yellow color produced upon cleavage was proportional to the amount of DEVDase activity.

### 2.7. Western blotting [33]

The celastrol-treated cells were lysed in a lysis buffer containing 20 mM Tris (pH 7.4), 250 mM NaCl, 2 mM EDTA (pH 8.0), 0.1% Triton X-100, 0.01 mg·mL-1 aprotinin, 0.005 mg·mL-1 leupeptin, 0.4 mM PMSF, and 4 mM NaVO4. The proteins were separated by SDS-polyacrylamidegel electrophoresis. After electrophoresis, the proteins were electrotransferred onto nitrocellulose membranes and blotted with various primary antibodies (1∶1000). Antibodies against Cyclin D1, P21, P27, Bcl-2, Bax, survivin, NF-kappa B p65, and internal control antibody GAPDH were obtained from EnoGene. The blot was washed, exposed to horseradish peroxidase-conjugated secondary antibodies for 1 h, and finally detected by ECL reagent (GE Healthcare). The band densitometric analysis of the scanned blots was performed using Image J software, and the results are expressed as fold change relative to the internal control.

### 2.8. Immunofluorescence [34]

The cells were grown on coverslips and cultured for 24 hours. After treatment with the indicated doses of celastrol, the cells were fixed in 4% paraformaldehyde for 15 minutes, washed in PBS, and treated for 15 minutes with PBS containing 0.1% Triton X-100. The cells were then washed, blocked with 10% bovine serum albumin (BSA) for 1 hour at room temperature, and incubated at 4°C overnight with NF-kappa B p65 antibody (1∶100) diluted in 0.1% BSA. After extensive washing, a 1∶200 dilution of FITC-conjugated goat anti-mouse immunoglobulin was applied as the secondary antibody for 1 hour at room temperature. Nuclear staining was achieved by incubating cells in DAPI for 5 minutes. The slides were then washed and photographed with an OLYMPUS 1X71 fluorescence microscope. Zinc Protoporphyrin-9 (ZnPPIX) is a potent, selective heme oxygenase inhibitor. In this study, 24 hrs before celastrol treatment, cells were treated with 1 µM Zinc Protoporphyrin-9 (Santa Cruz Biotechnology) to study if the effect of Celatrol on NF-kB is heme oxygenase-dependent.

### 2.9. Statistical analysis

All assays were repeated thrice to ensure reproducibility. The results are expressed as mean ± standard deviation (SD). The significance of results obtained from the control and treated groups was performed by Student's unpaired t-test and one way analysis of variance. Means and standard deviations were calculated. A probability (P) value less than 0.05 was considered statistically significant.

## Results

### 3.1. Celastrol induced proliferation inhibition in LP-1 MM cell

**Figure 1:**
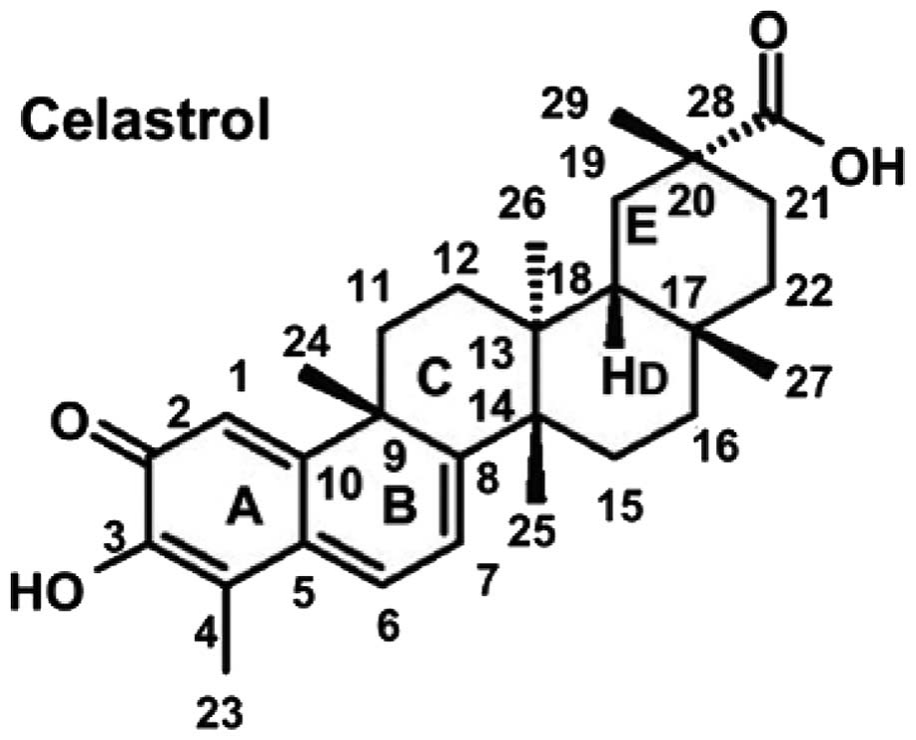
Chemical structures of Celastrol. Celastrol is a natural triterpenoid quinone methide isolated from the Chinese plant genuses of celastrus, maytenus, and tripterygium.

**Figure 2:**
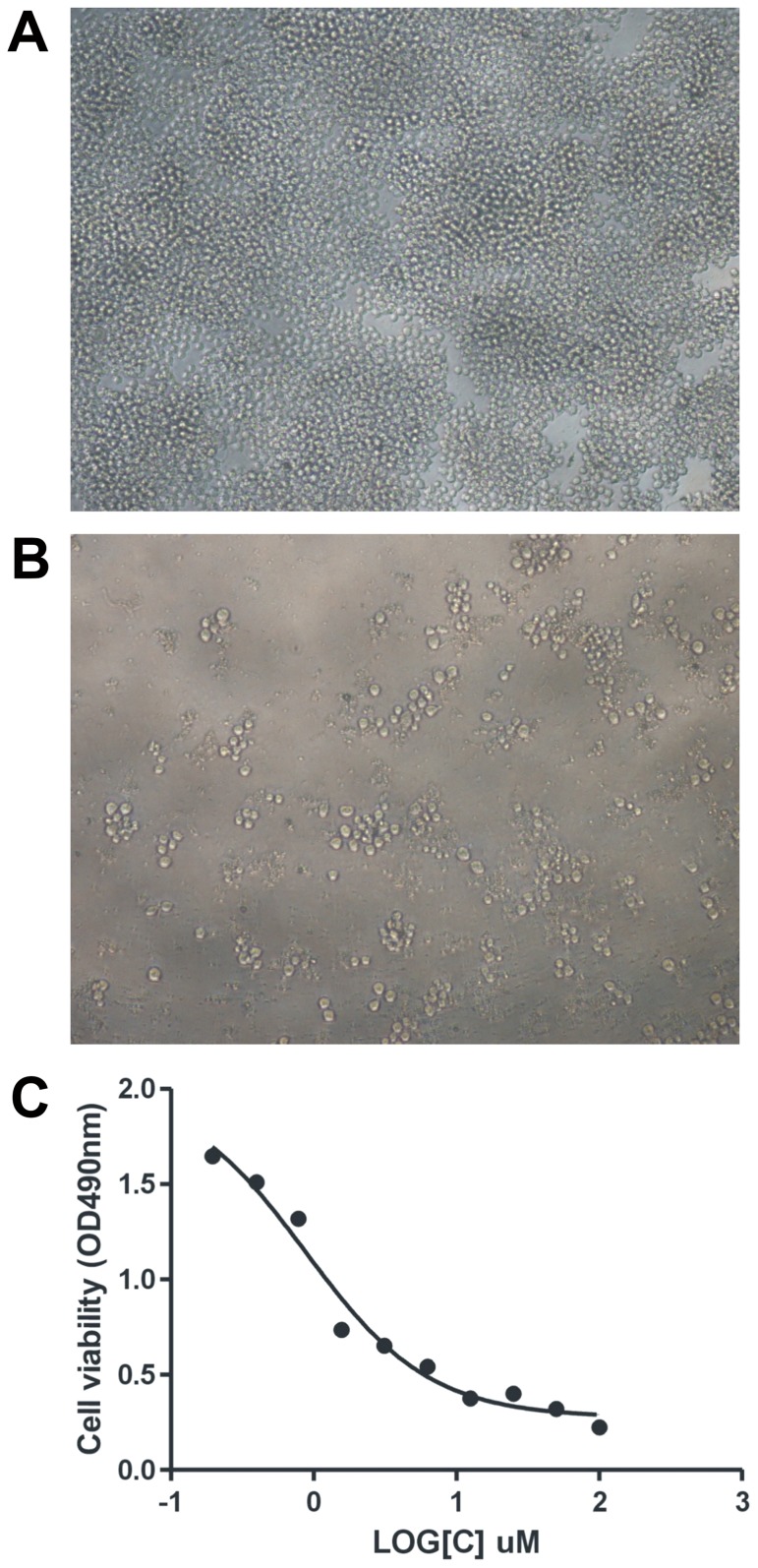
Celastrol inhibited LP-1 human multiple myeloma cell proliferation. Morphological signatures of celastrol growth inhibitory effects. Cells were treated with various doses of celastrol and cell viability was determined by MTT assay. Computer-assisted phase-contrast microscopy illustrations of control LP-1 human multiple myeloma cells (A) and treated with celastrol at concentration 0.78125 µM for 72 h (B). IC50 was shown as calculated by Prism 5.0. The IC50 growth inhibitory concentration for 72 h is 0.8817 µM (C).

The effects of celastrol was examined with triterpenoid quinone methide structure (Figure 1) on the cellular proliferation of human MM cell line LP-1. Celastrol inhibited cell proliferation of LP-1 MM cell in a dose-dependent manner with IC50 values of 0.8817 µM (Figure 2). At the concentration of 50 µM, the cell proliferation inhibitory rate was over 80%. Significant morphological changes such as shrinkage of cell and detachment of cell from the surrounding of LP-1 myeloma cell were observed at a concentration of 0.78125 µM.

### 3.2. Celastrol-induced cell proliferation inhibition was mediated through G1 cell-cycle arrest in LP-1 myeloma cells

**Figure 3:**
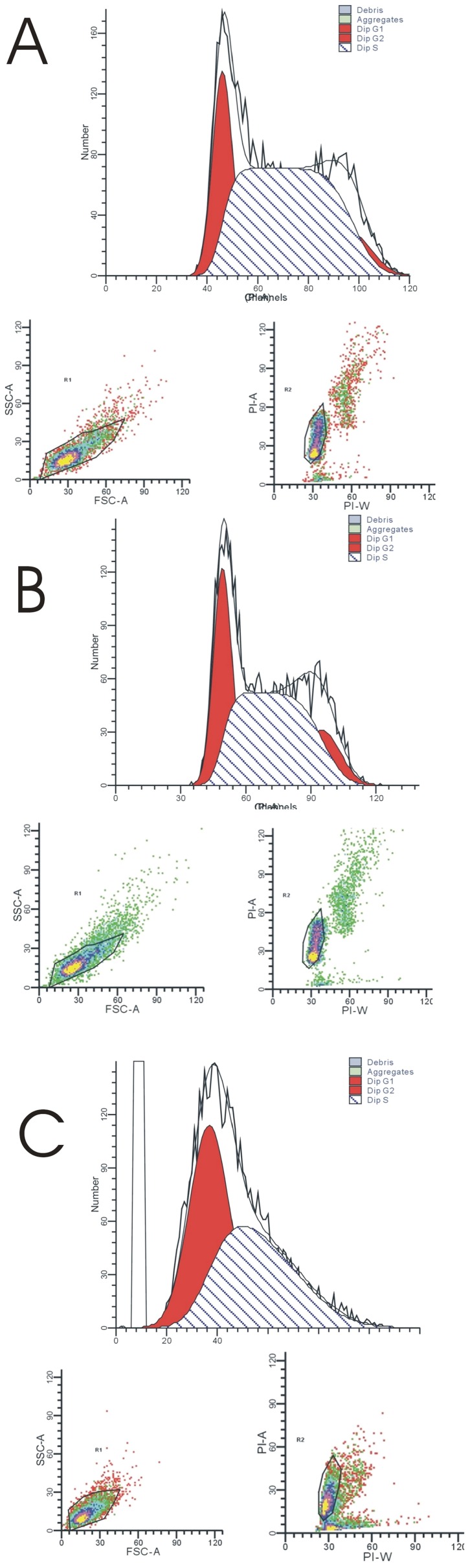
Celastrol causes accumulation of LP-1 human multiple myeloma cells in the G1 phase. LP-1 cells (2×106 mL-1) were treated with 0.5 µM celastrol for 0 (A), 12 (B) or 24 h (C), after which the cells were washed, fixed, stained with PI, and analyzed for DNA content by flow cytometry. Cell population in each cell cycle phase was numerically depicted. The G1 phase cell population was 24.15%, 29.79% and 49.20%, respectively. Data represent one of three independent experiments.

To investigate if the proliferation inhibition by celastrol was caused by cell cycle arrest of LP-1 myeloma cell, the cell cycle distribution of LP-1 cell treated with 0.5 µM of celastrol for 12 and 24 h was analyzed using flow cytometry. There was an increase in the cells in the G1 phase (Fig. 3), which suggested that celastrol induced G1 phase arrest.

### 3.3. p27 induction was essential for Celastrol induced cell cycle arrest in LP-1 MM cell

**Figure 4:**
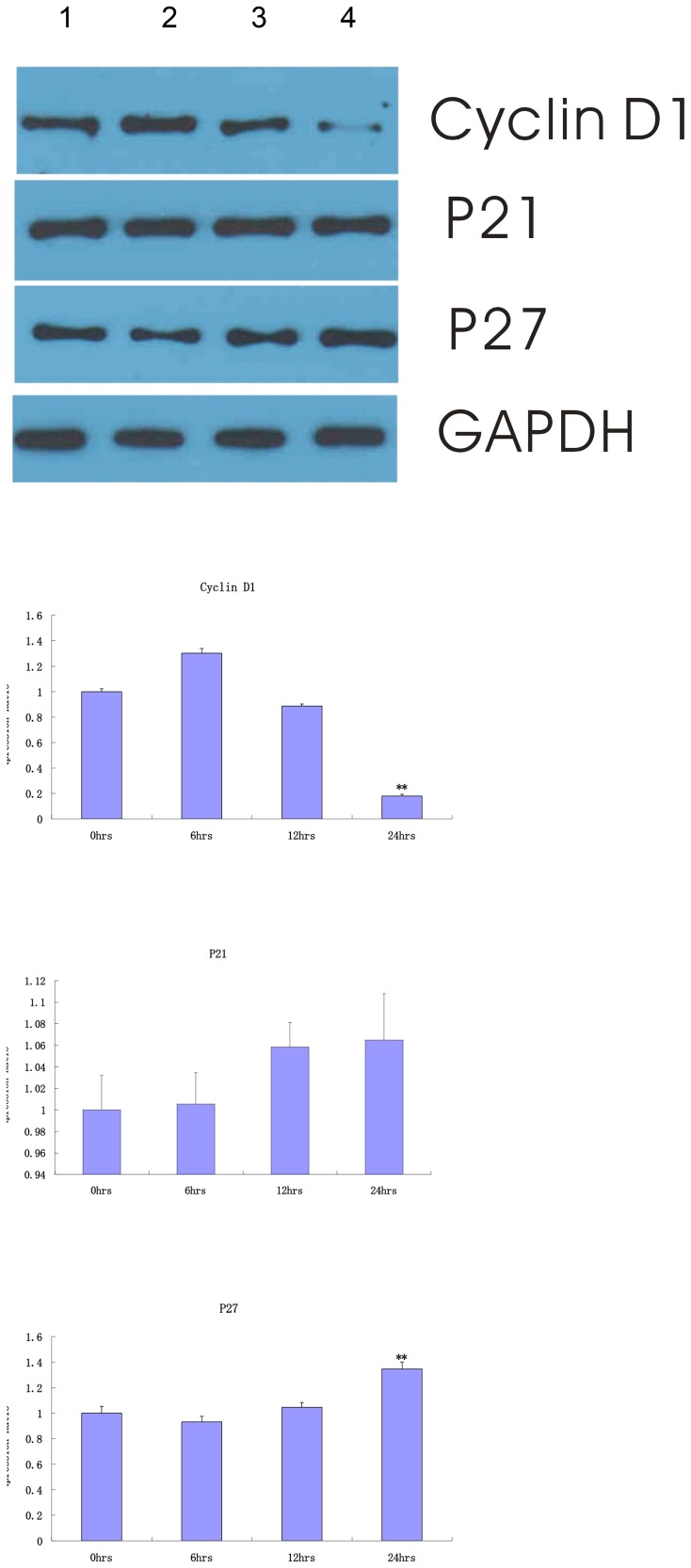
Celastrol up-modulated p27 protein expression in LP-1 cells. LP-1 cells were treated with 0.5 µM celastrol for 0 h (lane 1), 6 h (lane 2), 12 h (lane 3), and 24 h (lane 4), respectively. Whole cell lysates were prepared, separated on SDS-PAGE, and subjected to Western blot using antibodies against Cyclin D1, p27, and p21. The same blots were stripped and reprobed with GAPDH antibody to show equal protein loading. Expression fold was the ratio of protein expression in the celastrol-treated group to 0 h. Columns, mean; bars, SD (n = 3). *, p<0.05; **, p<0. 01.

The cell division relies on the activation of cyclins, which bind to the cyclin-dependent kinases (CDKs) to induce cell-cycle progression towards S phase and later to initiate mitosis. Since the uncontrolled CDK activity is often the cause of human cancer, their function is tightly regulated by cell-cycle inhibitors such as the p21 and p27 Cip/Kip proteins. Therefore, the cyclin D1, p21, and p27 Cip/Kip expression was investigated in LP-1 cell treated with 0.25, 0.5, and 1 µM of celastrol for 24 h. The study findings convincingly showed that an up-regulation of p27 but not p21 and down-regulation of cyclin D1 were the most important and central event in celastrol-induced G1 arrest in human myeloma cells (Figure 4).

### 3.4. Induction of apoptosis by celastrol in myeloma cells

**Figure 5:**
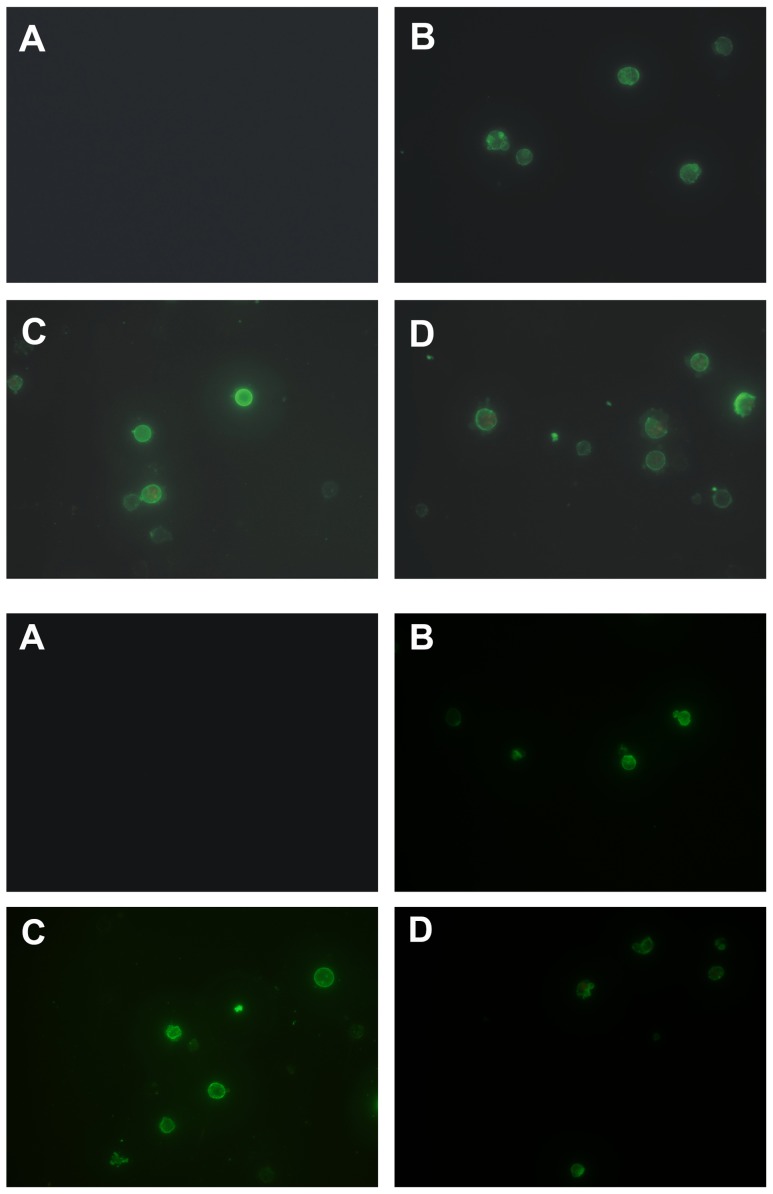
Celastrol induced apoptosis in LP-1 cells (up) and RPMI 8226 cells (down). Cells were treated with 1 µM celastrol for 0 h (A), 12 h (B), 24 (C), and 36 h (D) incubated with Annexin V conjugated with EGFP and analyzed with a fluorescence microscope for early apoptotic effects.

**Figure 6:**
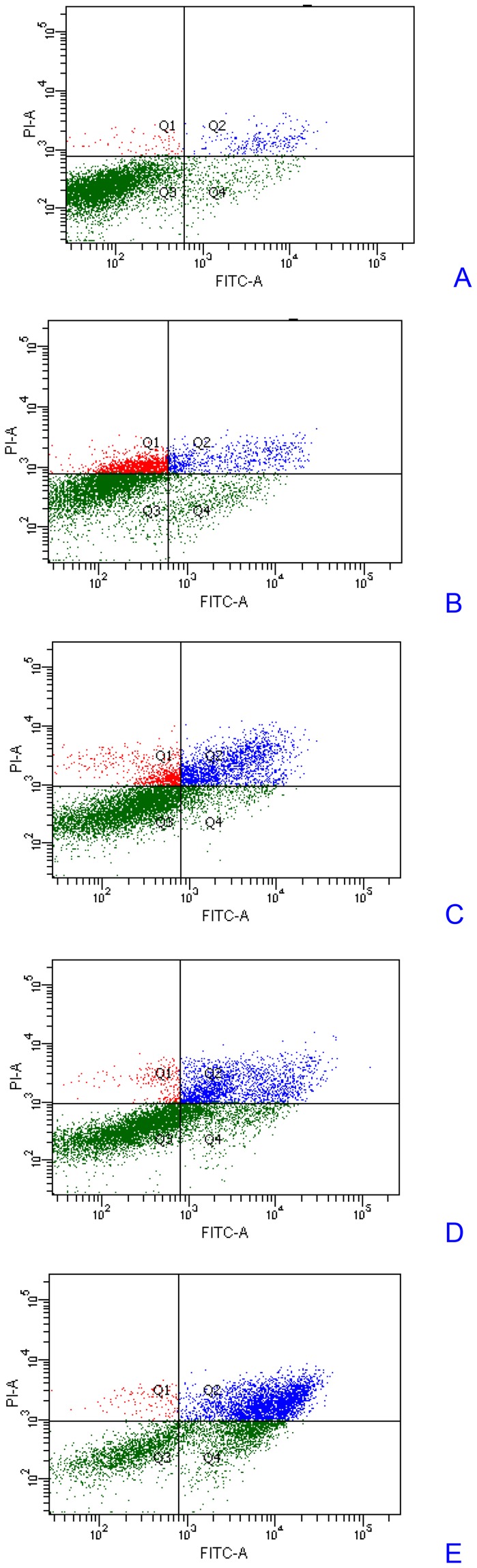
Celastrol induced apoptosis in a LP-1 cells. LP-1 cells were treated with 1 µM celastrol for 0 h (A), 12 h (B), 24 h (C), 36 h (D), and 48 h (E) incubated with Annexin V conjugated with EGFP and analyzed with a flow cytometer for apoptotic effects. The apoptotic cell percentage was 7.8±0.4%, 16.1±1.2% (p<0.05 vs 0 h), 18.0±3.1% (p<0.05 vs 0 h), 39.5±5.8% (p<0.01 vs 0 h), and 71.2±8.6% (p<0.01 vs 0 h), respectively. The results shown are representative of three independent experiments.

To investigate the mechanism of cytotoxic effect of celastrol, the apoptosis using Annexin-V and PI-double staining was measured in both LP-1 and RPMI 8226 cells to investigate that the apoptotic effect of celastrol is not specific to only LP-1 cells. An early indicator of apoptosis is the rapid translocation and accumulation of the membrane phospholipid phosphatidylserine from the cytoplasmic interface to the extracellular surface. This membrane asymmetry loss could be detected by utilizing the binding properties of Annexin V. Staining was tested by both fluorescence microscope (Figure 5) and flow cytometry (Figure 6). After treatment with 1 µM celastrol for 0 h, 12 h, 24 h, 36 h, and 48 h, the apoptotic LP-1 cell percentage was 7.8±0.4%, 16.1±1.2% (p<0.05 vs 0 h), 18.0±3.1% (p<0.05 vs 0 h), 39.5±5.8% (p<0.01 vs 0 h), and 71.2±8.6% (p<0.01 vs 0 h), respectively.

### 3.5. Caspase-3 activation was involved in celastrol induced apoptosis

**Figure 7:**
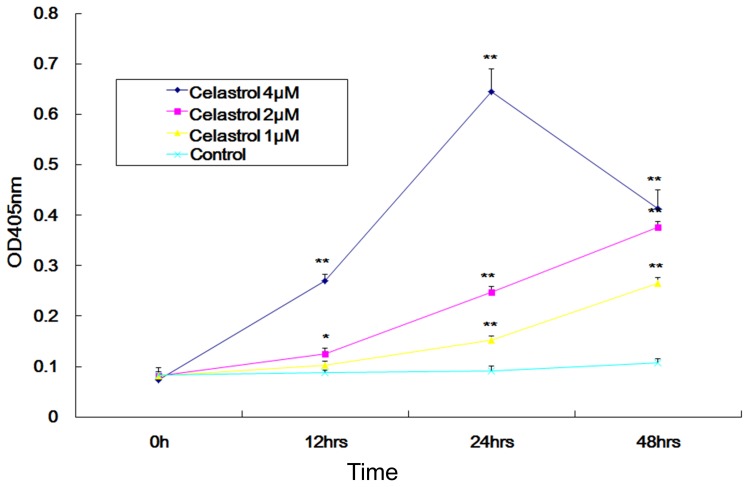
Celastrol induced caspase-3 activation. The cells were treated with 0 µM, 1 µM, 2 µM, and 4 µM celastrol for the indicated times. The whole cell extracts were prepared and subjected to enzymatic activity of caspase-3 by colorimetric substrate Ac-DEVD-pNA. The amount of yellow color at 405 nm indicating Caspase-3 activation fold was compared in the celastrol treated group to 0 h. point, mean; bars, SD (n = 3). *, p<0.05; **, p<0. 01.

Caspase-3 is a crucial mediator of apoptosis and it catalyzes the specific cleavage of many key cellular proteins. Therefore, the activity of caspase-3 in LP-1 cell treated with 1 µM, 2 µM, and 4 µM of celastrol for 0 h, 12 h, 24 h and 48 h were investigated. After 24 h treatement with 4 µM of celastrol, the caspase-3 activation fold was 8.7 times than that at 0 h. The activity of caspase-3 increased in a time-dependent and dosage-dependent manner (Figure. 7), suggesting that the caspase-3 pathway might play an essential role in celastrol-induced apoptosis in LP-1 MM cell.

### 3.6. Celastrol Down-modulated the Expression of Antiapoptotic Proteins

**Figure 8:**
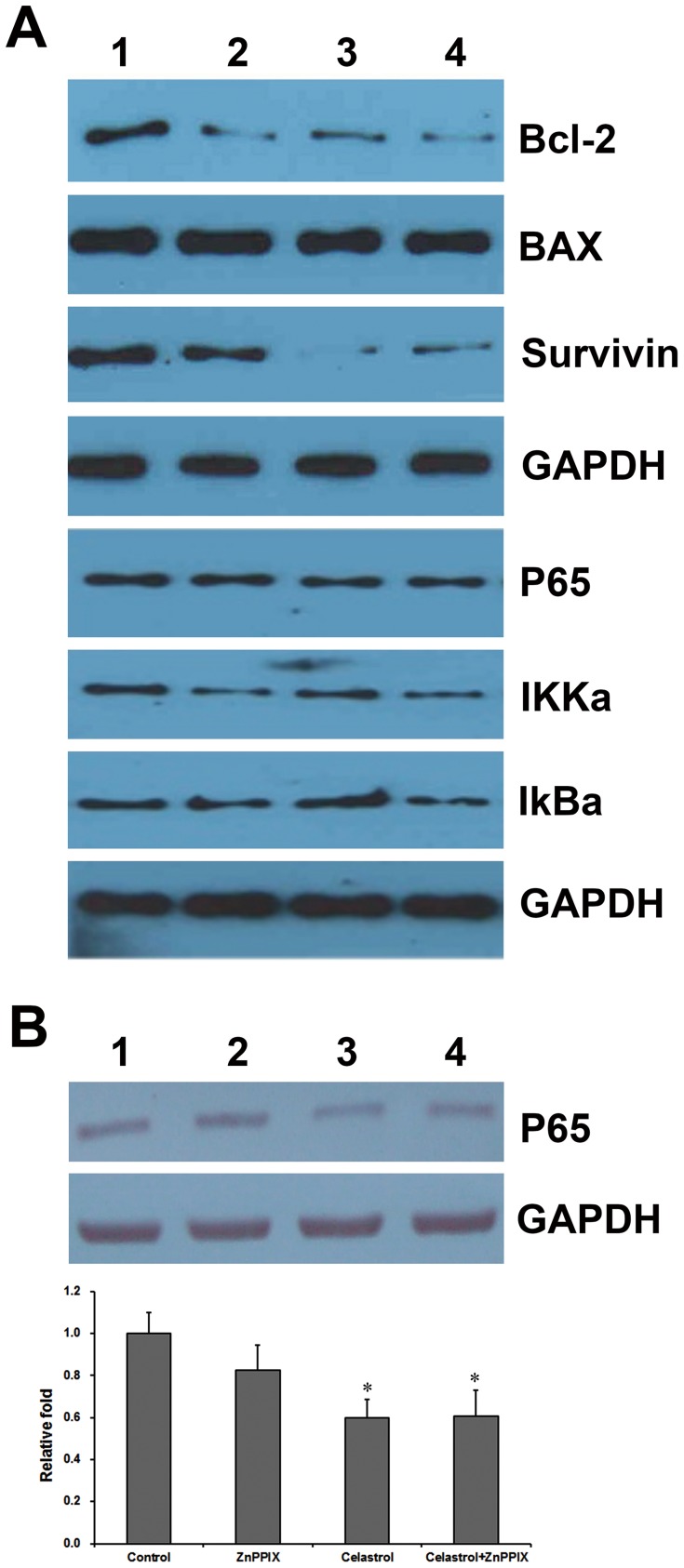
Celastrol down-modulated antiapoptotic proteins expression in LP-1 cells. (A). LP-1 cells were treated with 1 µM celastrol for 0 h (lane 1), 12 h (lane 2), 24 h (lane 3), and 48 h (lane 4), respectively. The whole cell lysates were prepared, separated on SDS-PAGE, and subjected to Western blot using antibodies against Bcl-2, Bax, Survivin, P65, IKKa, and IkBa. (B). LP-1 cells were treated with medium (lane 1), 1 µM ZnPPIX (lane 2), 1 µM celastrol (lane 3), and 1 µM celastrol combined with 1 µM ZnPPIX (lane 4) for 24 h, respectively. The same blots were stripped and reprobed with GAPDH antibody to show equal protein loading. Relative expression fold was first normalized by GADPH and then the ratio of protein expression in the celastrol/ZnPPIX -treated group was compared to control. Columns, mean; bars, SD (n = 3). *, p<0.05. No significance was observed from cells treated with Celastrol combined with ZnPPIX and those treated with only Celastrol (p>0.05).

The mechanism of celastrol induced apoptosis was investigated. LP-1 cells were treated with different concentrations of celastrol for 24 h and then examined for the expression of antiapoptotic proteins using relevant antibodies. The results indicated that celastrol down-regulated the expression of Bcl-2 and survivin (Figure 8A), whereas the down-regulation of Bax was less pronounced. The down-regulation of Bcl-2 was quite dramatic and was dose-dependent (Figure 8A).

### 3.7. NF-κB pathway is crucial in celastrol-induced apoptosis

**Figure 9:**
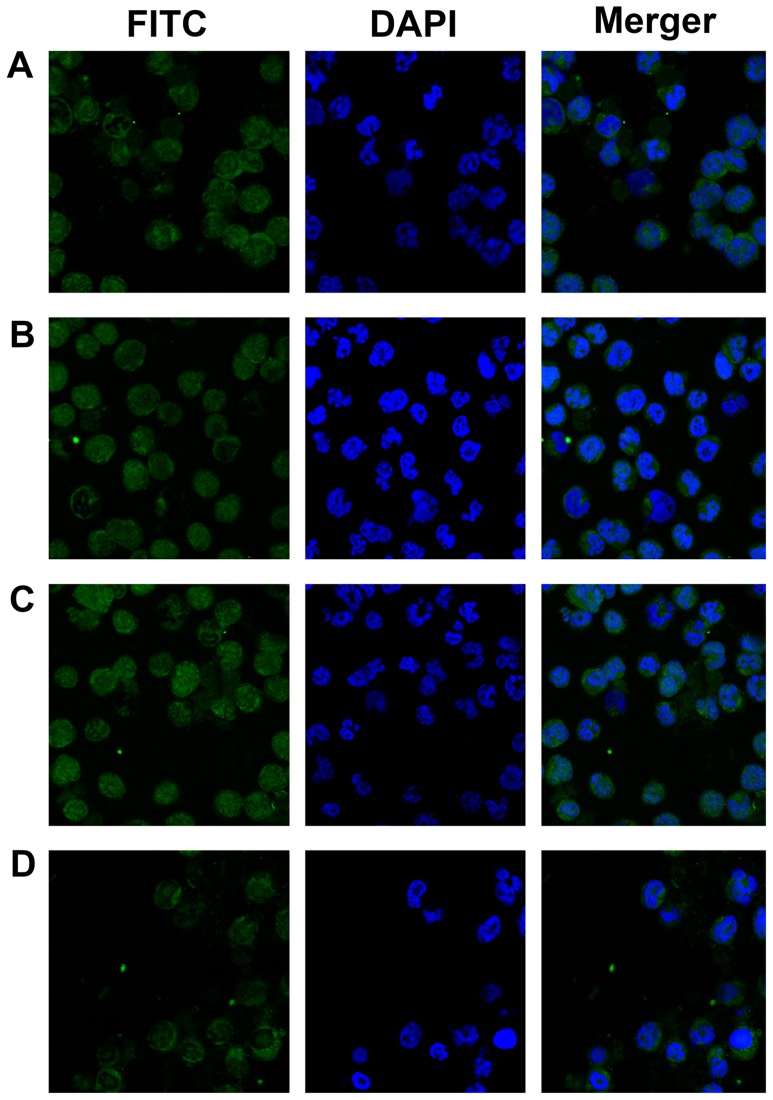
Celastrol inhibited constitutively active NF-kB in LP-1 MM cells. An altered subcellular distribution of NF-κb was observed. LP-1 cells were incubated with medium (A), 1 µM celastrol for 30 min (B), 1 µM Zinc Protoporphyrin-9 (ZnPPIX) (C), 1 µM celastrol combined with 1 µM ZnPPIX (D) and then analysed for the intracellular distribution of p65 by fluorescence microscope. Green indicates p65, and blue indicates nuclei (original magnification ×400). The results shown are representative of three independent experiments.

The NF-κB pathway is a key regulator of cytokine stimulation, cell cycle, apoptosis, and angiogenesis [35]. It is also critical in the progression and apoptosis of cancer cells including MM. Recently, the inhibition of NF-κB pathway using the proteasome inhibitor bortezomib was found to be pivotal in the treatment of untreated and relapse/refractory myeloma [36], [37]. Hence, the NF-κB pathway was examined in this study by Western blotting. The expression of NF-κB and IKKa were decreased after celastrol treatment (Figure 8A) and this effect was HO-1-independent by using ZnPPIX, a potent heme oxygenase inhibitor (Figure 8B). Nuclear translocation of the NF-kappa B p65 subunit was examined by immunofluorescence after treatment with celastrol. As shown in Figures 9A, 9B, celastrol effectively blocked nuclear translocation of the p65 subunit as well as IκB-α cleavage in a time-dependent manner, and this effect was also HO-1-independent by using ZnPPIX (Figure 9C, 9D).

## Discussion

MM is a clonal plasma cell malignancy with a clinical median overall survival of 3–5 years. The existing treatment modalities included thalidomide, lenalidomide, bortezomib, and autologous transplantation. All these target myeloma cells and their microenvironments and have shown remarkable activity against refractory prolonged the progression-free and overall survival of MM patients. However, the majority of patients eventually experiences a relapse and chemoresistance, and ultimately dies due to the disease.

The plants used in traditional Chinese medicine are rich sources of biologically active substances with potential therapeutic effects towards many human diseases. In this study, celastrol was found to inhibit the growth of human myeloma cell line LP-1 cells with an IC50 of <1 µM. In addition, celastrol induced G1 cell cycle arrest followed by cell apoptosis.

The cell division relies on the activation of cyclins, which bind to CDKs to induce cell-cycle progression towards S phase and later to initiate mitosis. Since the uncontrolled CDK activity is often the cause of human cancer, their function is tightly regulated by cell-cycle inhibitors such as the p21 and p27 Cip/Kip proteins. Following anti-mitogenic signals or DNA damage, p21, and p27 bind to cyclin-CDK complexes to inhibit their catalytic activity and induce cell-cycle arrest.

In the present study, the protein level of Cyclin D1 started decreasing at 0.5 µM celastrol for 24 h, and there was a significant Cyclin D1 decreased expression (p<0. 01). On the other hand, the celastrol treatment of cells for 24 h showed a strong increase in p27 protein level (p<0. 01), which further suggested a possible role of p27 in celastrol to cause G1 arrest. In the case of p21, its protein level remained sustained until 24 h. After an up-regulation, p27 negatively regulate the kinase activity of the CDKs that alters the phosphorylation of Rb protein, keeping it in hypophosphorylated form and bound to transcription factor E2Fs [38]. The Rb-bound E2Fs are inactive and therefore fail to activate their downstream target genes involved in the cell cycle progression and DNA replication.

Activation of NF-κB is initiated by the signal-induced degradation of IκB proteins [39]. This occurs primarily through the activation of IκB kinase (IKK). When activated by signals usually come from the outside of the cell, the IκB kinase phosphorylates two serine residues located in an IκB regulatory domain [40]. When phosphorylated on these serines (e.g., serines 32 and 36 in human IκBα), the IκB inhibitor molecules are modified by ubiquitination, which then leads them to be degraded by proteasome. The NF-κB pathway is a potential therapeutic target for cancer [39]. Proteasome inhibitor bortezomib, which inhibits NF-κB activation, has been widely been used to treat MM patients worldwide [41]. Therefore, the natural products that inhibit NF-κB activation could be the novel potential agents for the treatment of MM [42].

Celastrol functions as an active natural proteasome inhibitor, and the celastro-induced myeloma cell apoptosis is associated with NF-kB attenuation in vitro [43]. The present study finding suggests that the suppression of NF-kB by targeting proteasome in myeloma may be applicable in disrupting myeloma progression including its primary growth and metastasis. Although more evidence is needed to delineate the role of NF-kB in the celastrol-mediated myeloma regression, the current study reveals that celastrol inhibits multiple NF-kB-driven protein expression that is involved in myeloma proliferation.

In accordance with the previous reports, it was found that the MM cell lines expressed the constitutively activated NF-kB, and that celastrol suppressed this activation and nuclear translocation of NF-kappa B p65. Although celastrol has been shown to inhibit NF-kB activation in various tumour cell lines, how celastrol can inhibit the constitutively activated NF-kB in MM cell lines has not been previously studied. It was observed that celastrol suppressed constitutively p65 and IKK, which may led to the inhibition of phosphorylation of p65 and IkBa, respectively [44].

In the recent years, NF-kappa B activation has been linked to many aspects of tumorigenesis including the control of apoptosis, cell cycle, differentiation, cell adhesion, cell migration, and angiogenesis [45]. NF-kappa B regulates the expression of several genes whose products are involved in anti-apoptosis such as Bcl-xL, cIAP, and TRAF. Bcl-2 [46]. The finding that residual tumors at the completion of chemotherapy express increased levels of Bcl-2 compared with pretreatment specimens suggests that Bcl-2 expression might be one mechanism for tumor resistance [47]. In the present study, the levels of Bcl-2 were significantly decreased after exposure to celastrol, although there was little change in Bax in response to celastrol. In comparison, the activation of caspase-3 could easily be detected. It has been found that many chemotherapeutic agents (including taxol, doxorubicin, and etoposide) can activate NF-kappa B, which can prevent apoptosis, block the ability of therapeutic agents to induce cell death, and lead to resistance to apoptosis induced by chemotherapeutic agents.

It has been reported that in AML-derived cells, but not primary cells, Heme oxygenase-1 (HO-1) is upregulated in response to TNF stimulation in conjunction with NFκB inhibition [48]. Heme oxygenase-1 (HO-1) is an Nrf2 transcription factor-regulated gene that is commonly induced following oxidative stress and cellular injury, functioning to decrease oxidative stress and inflammatory responses, protecting against apoptosis and altering the cell cycle [49]. It has been reported that bortezomib increases HO-1 expression in a time- and concentration- dependent manner. Moreover, we also observe that HO-1 is increased in lenalidomide-resistant multiple myeloma cell lines [50].

The pivotal role of the NF-kappa B pathway in the inhibition of cell apoptosis strongly suggests that NF-kappa B inhibitors would be useful in cancer therapy. Our study has identified that the effect of Celatrol on NF-kB is HO-1-independent. It is highly possible that celastrol could overcome the apoptosis resistance of multiple myelome chemotherapy caused HO-1 and Nrf2 induction. Work is ongoing to study whether celastrol induced NF-kB inhibitory effect is through Nrf2-HO-1 pathway.

## Conclusion

The present study results show that celastrol may be used as a NF-kappa B inhibitor to inhibit myeloma cell proliferation.
